# OXA-204 Carbapenemase in Clinical Isolate of *Pseudomonas guariconensis*, Tunisia

**DOI:** 10.3201/eid3106.250131

**Published:** 2025-06

**Authors:** Nadia Jaidane, Wejdene Mansour, Lamia Tilouche, Pierre Châtre, Pauline François, Agnese Lupo, Saoussen Oueslati, Aymeric Jacquemin, Ali Lazzem, Sonia Karaborni, Laetitia Du Fraysseix, Yomna Ben Lamine, Nidhal Mahdhi, Abdelhalim Trabelsi, Jean-Yves Madec, Marisa Haenni, Thierry Naas

**Affiliations:** Université de Sousse Faculté de Médecine, Sousse, Tunisia (N. Jaidane, W. Mansour); Sahloul Hospital, Sousse (L. Tilouche); Anses Laboratoire de Lyon, Lyon, France (P. Châtre, P. François, A. Lupo, L. Du Fraysseix, J.-Y. Madec, M. Haenni); Inserm U1184, Le Kremlin-Bicêtre, France (S. Oueslati, A. Jacquemin, T. Naas); Sahloul University Hospital, Sousse (A. Lazzem, S. Karaborni, Y. Ben Lamine, N. Mahdhi, A. Trabelsi); Université Paris-Saclay BU Kremlin-Bicêtre, Le Kremlin-Bicêtre, France (T. Naas)

**Keywords:** *Pseudomonas* *guariconensis*, OXA-204, carbapenemase, CMY-16, burn unit, bacteria, bacterial infection, Enterobacterales, antimicrobial resistance, multidrug resistance, Tunisia

## Abstract

We report an OXA-204–producing *Pseudomonas guariconensis* clinical isolate in Tunisia, proving the spread of OXA-48 variants beyond Enterobacterales. The *bla*_OXA-204_ gene was carried on a 119-kb chromosomally integrated plasmid fragment, along with multiple additional resistance genes. Surveillance, diagnostic tools, and antimicrobial drug access are needed to mitigate spread of carbapenem-resistant pathogens.

Multidrug-resistant (MDR) *Pseudomonas* spp. are major contributors to life-threatening infections, especially in severe burn patients. *Pseudomonas guariconensis* was initially isolated from rhizospheric soils in 2013 (*1*) but has since been described in various clinical contexts, underscoring its pathogenic potential. Clinical manifestations of *P. guariconensis* infections include infective endocarditis (*2*), necrotizing fasciitis (*3*), and asymptomatic bacteriuria with a VIM-2 metallo-β-lactamase–producing isolate (*4*). We characterized the molecular mechanism sustaining carbapenem resistance in a clinical *P.* *guariconensis* isolate from Tunisia.

## The Study 

On May 28, 2023, an 8-year-old child with severe high-voltage electric shock burns on 64% of his body was admitted to the Center for Traumatology and Major Burns, Ben Arous (Tunis, Tunisia). Multiple complications developed, including sepsis, urinary tract infection, and wound infections, which required the administration of broad-spectrum antimicrobial drugs (cefotaxime, imipenem, gentamicin, colistin, teicoplanin, and fosfomycin). On August 25, 2023, we transferred the patient to the burn unit of Sahloul Hospital (Sousse, Tunisia), where he received nutritional support with a high-protein high-calorie diet, transfusions for anemia, and extensive wound care and surgical skin grafting procedures. On September 20, 2023, after we isolated *Morganella morganii*, *Proteus mirabilis*, methicillin-susceptible *Staphylococcus aureus*, and glycopeptide-resistant *Enterococcus faecium* from bone fragments, the patient received piperacillin/tazobactam and cotrimoxazole for 21 days. On October 12, 2023, positive blood cultures revealed an extremely drug-resistant gram-negative bacillus we identified as *Pseudomonas aeruginosa* by Vitek 2.0 (bioMerieux, https://www.biomerieux.com). We conducted matrix-assisted laser desorption/ionization time-of-flight mass spectrometry (Bruker Daltonics, https://www.bruker.com) and identified the isolate as *P. guariconensis* with a score of 1.84.

We determined the MICs of the *P. guariconensis* isolate (65411) by using broth microdilution (Sensititre; ThermoFisher, https://www.thermofisher.com) and interpreted according to clinical breakpoints of the Comité de l’Antibiogramme de la Société Française de Microbiologie/European Committee on Antimicrobial Susceptibility Testing referential (version 2024; https://www.sfm-microbiologie.org/wp-content/uploads/2024/06/CASFM2024_V1.0.pdf). MIC testing revealed resistance to nearly all antimicrobial drugs tested, including ceftolozane/tazobactam (MIC >16 mg/L), imipenem and meropenem (MIC >32 mg/L), imipenem/relebactam (MIC >16 mg/L), meropenem/vaborbactam (MIC >32 mg/L), and aztrenoam/avibactam (MIC >16 mg/L), but susceptibility to ceftazidime/avibactam (MIC <0.5 mg/L), cefiderocol (MIC <1 mg/L), and colistin (MIC <0.5 mg/L). We adjusted the treatment regimen on the basis of antimicrobial drug susceptibility results and the availability of medications in Tunisia, where cefiderocol and ceftazidime/avibactam are not yet available. We administered colistin (150,000 units/kg; 1 million units 3×/d intravenously for 25 days), along with rifampicin (20 mg/kg/d; 450 mg 1×/d intravenously for 15 days), resulting in clinical stabilization and improvement.

We reported a negative result when testing *P. guariconensis* 65411 by using a homemade carbaNP assay (*5*) and a positive result for OXA-48–like enzymes by using the lateral flow immunoassay NG-Test Carba 5 assay (NG-Biotech, https://www.ngbiotech.com) (*6*). We conducted short-read (Illumina, https://www.illumina.com) and long-read (Oxford Nanopore, https://nanoporetech.com) whole-genome sequencing to consolidate bacterial identification (we confirmed *P. guariconensis*-like-3 by using Centrifuge [*7*]), identify resistance genes (Center for Genomic Epidemiology, https://www.genomicepidemiology.org), and determine the *bla*_OXA-48-like_ genetic environment. We generated 1 contig of 5.6 Mbp with a guanine-cytosine content of 64.1% from a hybrid assembly by using Unicycler 0.5.0 (*8*), which agreed with the characteristics of reference *P. guariconensis* isolate ASM4082203v1 (GenBank accession no. GCF_040822035.1). We submitted the genome of isolate 65411 to the National Center for Biotechnology Information Nucleotide database (https://www.ncbi.nlm.nih.gov/nuccore; Bioproject no. PRJNA1150136).

The resistome of *P. guariconensis* 65411 contained acquired genes conferring resistance to β-lactams (*bla*_CMY-16_, *bla*_DHA-1_, *bla*_OXA-1_, *bla*_OXA-10_, and *bla*_OXA-204_), aminoglycosides [*aac(6')Ib-cr, ant(2'')-Ia*, *aph(3'')-Ib*, *aph(3')-Via*, *aph(3')-VIb*, *aph(6')-Id*], fosfomycin (*fosA*), fluoroquinolones [*aac(6')Ib-cr*], and additional antimicrobial drugs and biocides *(catB3*, *ARR-3*, *sul1/2*, *qacE*). High-level resistance to fluoroquinolones was because of a T83I substitution in the quinolone resistance-determining regions of GyrA. The RAST annotation identified an additional chromosome-encoded class A β-lactamase gene, named *bla*_GUA-1_, displayed 100% sequence identity with a ß-lactamase encoded in the genome of a *P. guariconensis* from the Czech Republic (GenBank accession no. UQM99659.1); 72% with that of *P. fulva* (accession no. MBF8778391.1), *P. mosselii* (accession no. WP_345894069.1), and *P. soli* (accession no. UXZ44560.1); and 57% with the extended-spectrum β-lactamase *bla_LUT-1_* from *P. luteola* (*9*). All conserved motifs essential for β-lactamase activity were preserved, including the serine active site motif (S_70_XXK), the hydrolytic water-binding site (S_130_DN), and the catalytic triad component (K_234_TG) (*10*). Those findings suggest the *bla*_GUA-1_ gene likely encodes an intrinsic class A β-lactamase (Appendix Figure). We cloned *bla*_GUA-1_ into an expression vector. The overexpression revealed crude protein extracts with strong nitrocefin hydrolytic activity. We confirmed the presence of a β-lactamase capable of hydrolyzing first- and third-generation cephalosporins (Appendix reference 1) by using ß-lactamase testing (Bio-Rad Laboratories, https://www.bio-rad.com).

**Figure 1:**
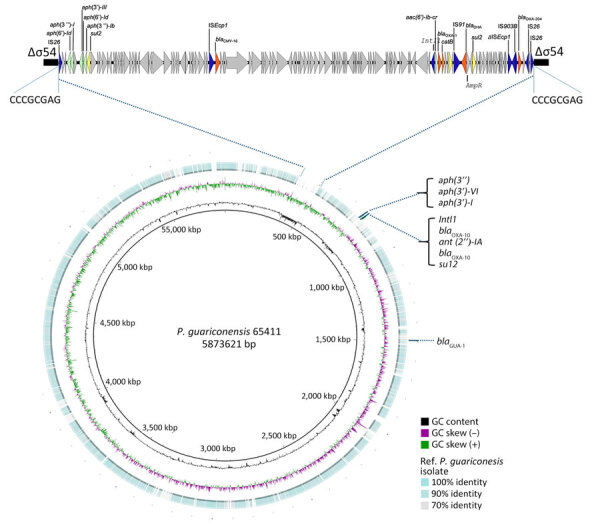
Comparative genomic analysis of OXA-204 carbapenemase-positive *Pseudomonas guariconensis* isolated from a child in Tunisia, 2023, using *P. guariconensis* GenBank accession no. GCF_040822035 as the reference genome. The concentric ring represents the BLAST (https://blast.ncbi.nlm.nih.gov) results of the *P. guariconensis* isolate from this study, 65411, with the reference genome. The color intensity of the outer ring indicates the level of sequence similarity, with darker shades representing higher sequence identity percentages. Gaps in the outer ring indicate very low identity or absence of the region. The magnified segment is the site of the IS*26*-mediated insertion of a 119-kbp plasmid fragment in the chromosome of *P. guariconensis* 65411. In the magnified portion, resistance genes and the associated mobile elements likely involved in their mobility are indicated in blue. Genes involved in resistance to β-lactams are indicated in orange (β-lactamases), to aminoglycosides in light green, to chloramphenicol in orange, and to sulfonamides in yellow. GC, guanine-cytosine. Figure created using BRIG software, https://sourceforge.net/projects/brig.

**Figure 2:**
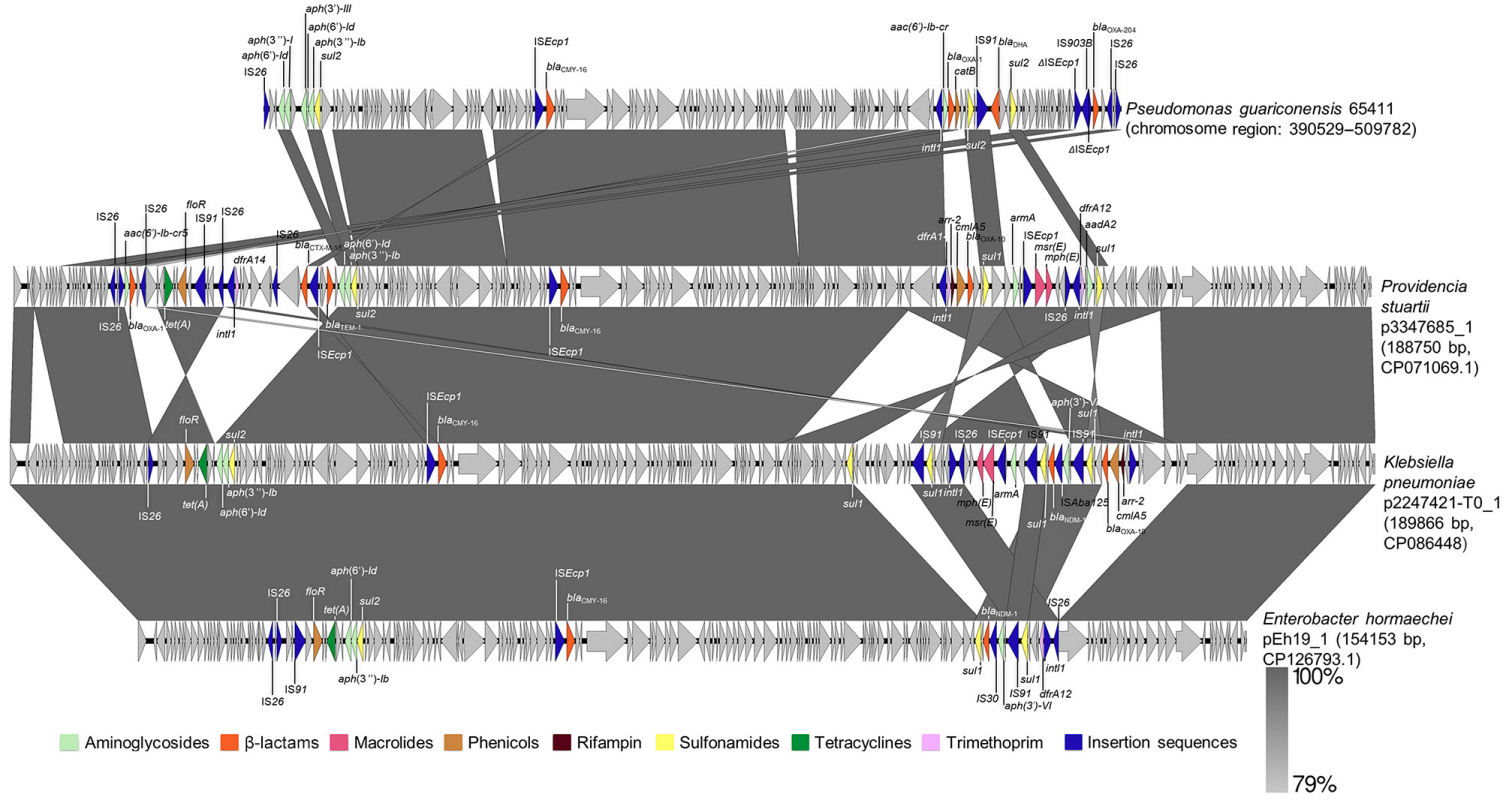
Representation of nucleotide alignment between the IS*26*-mediated transposon in the OXA-204 carbapenemase positive *Pseudomonas guariconensis* isolated from a child in Tunisia, 2023 (65411), and reference plasmids characterized in Enterobacterales (GenBank accession numbers provided). The matches ranged from 85% to 89% coverage, with 100% nucleotide identity. Arrows indicate genes and their transcription orientations. Open reading frames are colored according to antimicrobial drug family. Image created using Easyfig v.2.2.5 (https://mjsull.github.io/Easyfig).

**Figure 3:**
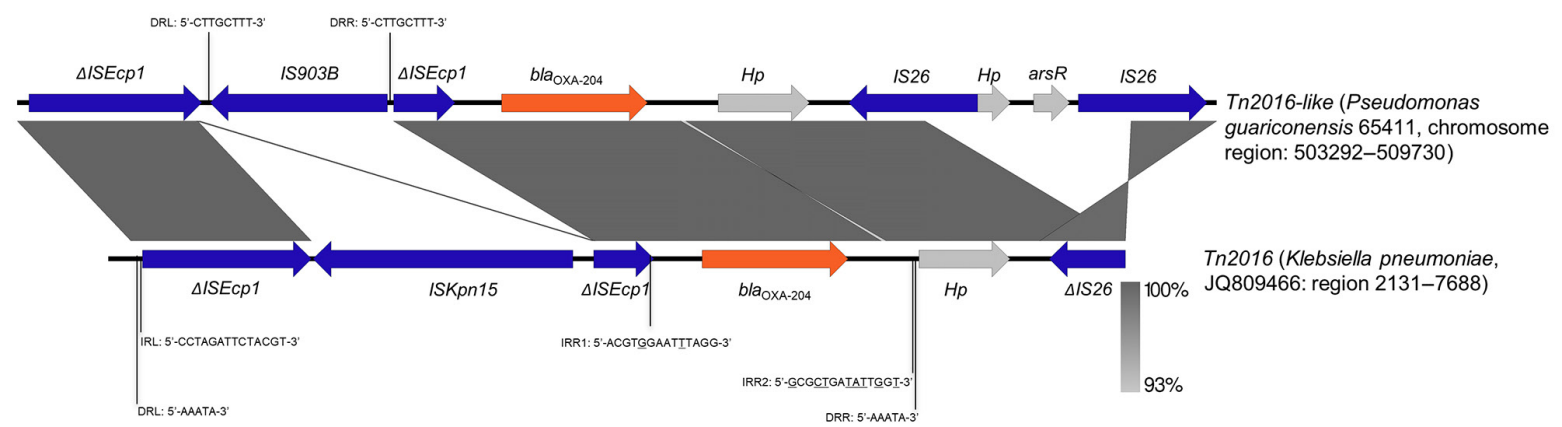
Representation of the nucleotide alignment between the Tn*2016*-like transposon carrying the *bla*_OXA-204_ gene in the OXA-204 carbapenemase positive *Pseudomonas guariconensis* isolated from a child in Tunisia, 2023 (65411), chromosome region 503292–509730, and the Tn*2016* characterized in *Klebsiella pneumoniae* plasmid p204B (GenBank accession no. JQ809466). Arrows indicate genes and their transcription orientations. DRL, direct repeats left; DRR, direct repeats right; Hp, hypothetical protein; IRL, inverted repeat left; IRR, inverted repeat right. Image created using Easyfig v.2.2.5 (https://mjsull.github.io/Easyfig).

The *bla*_OXA-204_ carbapenemase gene is mostly detected in Tunisia (*11*) and in France from patients with previous travel to Tunisia. The gene is mostly found in *Klebsiella pneumoniae* and *Escherichia coli* isolates (*11*; Appendix reference 2), but also rarely in *P. mirabilis*, *Citrobacter freundii*, *Serratia marcescens* (*12*), *Enterobacter cloacae* (*13*), and *Shewanella xiamenensis* (*14*). The *bla_OXA-204_* gene is described as part of a Tn*2016* transposon located on IncA/C plasmids (Appendix reference 1). In *P. guariconensis* 65411, the comprehensive analysis of the genetic environment of the *bla*_OXA-204_ gene revealed the integration of a 119-kb plasmid fragment into the chromosome (Figure 1). This fragment displayed multiple resistance genes, including the β-lactamases *bla_DHA-1_*, *bla_CMY-16_*, *bla_OXA-1_*, and *bla_OXA-204_*, alongside genes conferring resistance to chloramphenicol (*catB3*) and aminoglycosides [*aph(6')-Ic, aph(6')-Id, aph(3'')-I, aph(3')-III, aph(3')-IV, aph(3')-VI, aph(3')-VII,* and *aac(6')-Ib-cr*]. The inserted fragment was bound by 2 IS*26* elements and an 8-bp target site duplication, suggesting an IS*26*-mediated insertion into the chromosome. The inserted plasmid shared homology (100% homology over an 88% coverage of the 119-kbp fragment) with part of a 189,866-bp IncC plasmid originating from a *K. pneumoniae* isolate (GenBank accession no. CP086448) coproducing OXA-10, CMY-16, and NDM-1 (*15*) (Figure 2). The *bla_OXA-204_* gene was embedded within a 3,958 bp IS*Ecp1*-based Tn*2016*-like transposon (*11*; Appendix reference 1) bracketed by a 5-bp target site duplication (Figure 3). In the prototypical Tn*2016*, IS*Ecp1* was disrupted by an IS*Kpn15* (*11*; Appendix reference 1), whereas in *P. guariconensis* 65411, it was disrupted by an IS*903B* generating a 9-bp target site duplication in the Tn*2016*-like transposon. As suggested for IS*Kpn15* insertion, the IS*903B* insertion should not interfere with the strong outward oriented promoter of IS*Ecp1* (*11*; Appendix reference 1), enabling high-level expression of *bla*_OXA-204_.

## Conclusions

We describe an OXA-48-like carbapenemase in *Pseudomonas* clinical isolate, demonstrating the dissemination of *bla*_OXA-48-like_ genes beyond Enterobacterales. OXA-204–producing *P. guariconensis* was initially identified as *P. aeruginosa* by using biochemical methods, suggesting the real occurrence of *P. guariconensis* might be underestimated in clinical settings. The *bla*_OXA-204_ gene was carried by an IS*Ecp1*-based Tn*2016*-like element present on a 119-kb plasmid fragment inserted into the chromosome by a large IS*26*-mediated composite transposon. The integrated DNA carried additional resistance determinants, including 2 cephalosporinases (CMY-16 and DHA-1) and multiple associated resistance genes, resulting in an extremely drug-resistant phenotype. At first, the *bla*_OXA-204_ gene was characterized on a 150-kb IncA/C broad host range carrying *bla*_CMY-4_ that could be transferred to *P. aeruginosa* and lead to MICs for imipenem of 8 mg/L and meropenem of 32 mg/L (*11*). The high level carbapenem-resistance in *P. guariconenesis* 65411 is likely the result of the expression of OXA-204 carbapenemase together with CMY-16 and DHA-1, which are 2 cephalosporinases known to weakly hydrolyze carbapenems (Appendix reference 3).

Our findings reinforce the critical need for ongoing surveillance, advanced diagnostic tools, antimicrobial drug stewardship, and access to novel antimicrobial drugs in low- to middle-income countries to mitigate the spread of carbapenem-resistant pathogens. Carbapenemase detection should not rely exclusively on carbapenem-hydrolytic activity such as Carba NP but also on lateral flow immunoassays for confirming the presence of carbapenemases, including OXA-48–like enzymes in nonfermenting gram-negative pathogens.

Appendix Additional information about OXA-204 carbapenemase in clinical isolate of *Pseudomonas guariconensis*, Tunisia.
