# Supplementary material for: OXA-204 Carbapenemase in Clinical Isolate of Pseudomonas guariconensis, Tunisia
Source: Emerg Infect Dis. 2025 Jun;31(6):1197–201. doi: 10.3201/eid3106.250131 (PMC12123930; doi:10.3201/eid3106.250131)
Supplement: Appendix — Additional information about OXA-204 carbapenemase in clinical isolate of Pseudomonas guariconensis, Tunisia. [file 25-0131-Techapp-s1.pdf]

Article DOI: https://doi.org/10.3201/eid3106.250131

EID cannot ensure accessibility for supplementary materials supplied by authors. Readers who have difficulty accessing supplementary content should contact the authors for assistance.

OXA-204 Carbapenemase in Clinical Isolate of *Pseudomonas guariconensis*, Tunisia

Appendix

```
LUT-1 --------MNVILNRRTFLLASAVVSASYSLGTLAGANRDDASFQDRLAKLEQQLNGRLGVCAIDTANGAQLGYRANERFAMNSTFKVMLASAFLARSQDEPGLLEERLTYTRADLVTYS   112
GUA-1 --------MKPTFSRRQLIQGAMGGSV-----MLATPAWASDPVHQRLQHLEQALGGTLGLWALDTGSGRRLSYRSEARAALCSTFKVVLAAAILQQDQAEPGLLERRIAYTAEQLVVYS   107
FUL-1 ---MGSAHVKPTIRRRQLLQGILGCTS-----LLALPSWAADPLEQRLQRLERSADRTLGLWALDTGSGQQLAWRAEERFAFCSTFKAILAGAILHRSQAEPGLLERRIAYGAEQLVVYS   112
SOL-1 --------MTPTLSRRRVLQGALGCST-----LLALPTWADDPLRKALQQLEATTGSTIGLWALDTGSGRQLAWRGEERFAFCSTFKAVLAGAVLHRNEAEPGLLARRIAYGAEQLVVYS   107
MOS-1 --------MISTLSRRRVLQGALGCSA-----LLTLPAWAHDPLRQAMQQLEAASGSTIGLWALDTGSGRQLAWRGEERFAFCSTFKAVLAGAVLHRNEAEPGLLARRIAYGAEQLVVYS   107
              :   : ** .: .    :       *:        ... : :**   .  :*: *:**..* :*.:*.: * *: ****.:**.*.* : : :**** .*:.*   .**.**

LUT-1 PVTEKHVSTGMTVAELCAAGIQYSDNTAANVLMKKLGGPEAVTAFARSIGDTHFRLDRWETELNSAIPGDPRDTSTPQAMAMSLQRLALGDMLAADKQHQLQAWLKGNTTGGKRIRAGVP   232
GUA-1 PVTEKHVATGMSVAELCAAALQYSDNTAANLLLGVVGGPSKLTAYARSIGDPAFRLDRYEPALNSALPNDPRDTSTPLAMGVTLQRLLLTDGLPAAARLRLQDWLKGNTTGDTRIRAGLP   227
FUL-1 PVTQKHVGAGMTVAELCAAAVQYSDNTAGNLLLEQVGGPAGLTAFARRLGDPVLRLDRYEPELNSALPGDPRDTSSPLAMGATLERLLLTDGLPIAAQQQLQAWLKGNTTGATRIRAGLP   232
SOL-1 PVTEKHVGAGMTVAGLCAAAVQYSDNTAGNLLLELVGGPSGLTAFTRSLGDPTFRLDRNEPTLNTALPGDPRDTTTPLAMGVTLQRLLLGDGLAAAERQQLQDWLKGNTTGDARIRAGVP   227
MOS-1 PVTEKHVGPGMTVAELCAAAVQYSDNTAGNLLLELVGGPPGLTAFVRGLGDPTFRLDRNEPTLNTALPGDPRDTTTPLAMGVTLQRLLLTDGLPAGAQQQLQAWLKGNTTGDARIRAGLP   227
      ***:***. **:** ****.:*******.*:*:  :***  :**:.* :**  :**** *  **:*:*.*****::* **. :*:** * * *    : :** ********  *****:*

LUT-1 AGWQVGDKTGTGDYGSANDVAILWPPRRAPVVLALYSALENQQAEARNDVLADAARIVAEWVTG   296
GUA-1 EGWVVGDKTGSGDYGVANDVGIIWPPGQAPWILAIYTRGRDKTSPWRSDVIAEATRVVVEAWKV   291
FUL-1 EGWTVGDKTGSGDYGVANDVGIVWPPGRAPWILAVYSRGREKAAPANSELIAAATRVVVETWMA   296
SOL-1 KDWVVGDKTGSGDYGVANDVAVIWPKGRAPWIVVVFSRGTKADAPWNSETIAAATRVVVEAWKG   291
MOS-1 KDWVVGDKTGSGDYGVANDVAVIWPKGRAPWILVVFSRGTKQDAPWNSETIAAATRVVVEAWKG   291
       .* ******:**** ****.::**  :** ::.:::   .  :  ..: :* *:*:*.*
```

Appendix Figure. Amino acid sequence alignment of Gua-1 from *P. guariconensis* 65411 and *P. guariconensis* (UQM99659.1), Ful from *P. fulva* (MBF8778391.1), Sol from *P. soli* (UXZ44560.1), Mos from *P. mosselii* (WP_186595297.1) and Lut-1 from *P. luteola* (AAU10324.1). Conserved motives among class A beta-lactamases are highlighted in grey. The Omega loop is underlined.

Appendix References

1. Oueslati S, Iorga BI, Tlili L, Exilie C, Zavala A, Dortet L, et al. Unravelling ceftazidime/avibactam resistance of KPC-28, a KPC-2 variant lacking carbapenemase activity. J Antimicrob Chemother. 2019;74:2239–46. PubMed https://doi.org/10.1093/jac/dkz209

2. Charfi K, Mansour W, Khalifa AB, Mastouri M, Aouni M, Mammeri H. Emergence of OXA-204 β-lactamase in Tunisia. Diagn Microbiol Infect Dis. 2015;82:314–7. PubMed https://doi.org/10.1016/j.diagmicrobio.2015.04.003

3. Mammeri H, Guillon H, Eb F, Nordmann P. Phenotypic and biochemical comparison of the carbapenem-hydrolyzing activities of five plasmid-borne AmpC β-lactamases. Antimicrob Agents Chemother. 2010;54:4556–60. PubMed https://doi.org/10.1128/AAC.01762-09
